# The association between self-reported varenicline adherence and varenicline blood levels in a sample of cancer patients receiving treatment for tobacco dependence

**DOI:** 10.1016/j.abrep.2018.06.006

**Published:** 2018-07-04

**Authors:** Grace Crawford, Nancy Jao, Annie R. Peng, Frank Leone, Ravi Kalhan, Rachel F. Tyndale, Jessica Weisbrot, Brian Hitsman, Robert Schnoll

**Affiliations:** a Department of Psychiatry, Perelman School of Medicine, University of Pennsylvania, 3535 Market Street, 4th Floor, Philadelphia, PA 19104, USA; b Department of Preventive Medicine, Feinberg School of Medicine, Northwestern University, 680 North Lake Shore Drive, Suite 1400, Chicago, IL 60611, USA; c Department of Pharmacology & Toxicology, University of Toronto, 1 King's College Circle, Toronto, ON M5S-1A8, Canada; d Department of Medicine, Perelman School of Medicine, University of Pennsylvania, 51 N. 39th Street, Suite 251 Wright-Saunders Building, Philadelphia, PA 19104, USA; e Department of Medicine, Feinberg School of Medicine, Northwestern University, 676 North Saint Clair Street, Suite 1400, Chicago, IL 60611, USA; f Campbell Family Mental Health Research Institute, Centre for Addiction and Mental Health and Division of Brain and Therapeutics, Department of Psychiatry, University of Toronto, 250 College Street, Toronto, ON M5T-1R8, Canada; g Department of Psychiatry and Abramson Cancer Center, Perelman School of Medicine, University of Pennsylvania, 3535 Market Street, Philadelphia, PA 19104, USA

**Keywords:** Adherence, Varenicline, Smoking cessation, Tobacco dependence

## Abstract

**Introduction:**

The degree to which smokers quit successfully with varenicline is strongly associated with their adherence to the medication regimen. Thus, measuring varenicline adherence to identify smokers needing additional intervention is a priority. Few studies, however, have examined the validity of self-reported varenicline adherence, using a biological assessment of adherence as a reference. No study has examined this issue among cancer patients trying to quit smoking, who may show unique patterns of adherence given their medical comorbidity.

**Methods:**

This study used data from 76 cancer patients who received varenicline and provided self-reported varenicline adherence data (pill count) and a blood sample to determine varenicline metabolites 4 weeks after initiating varenicline.

**Results:**

Receiver operating characteristic (ROC) curve analyses of plasma varenicline levels showed that 4 ng/ml was the optimal cut-point for differentiating adherence with significant (*p*'s < 0.04) area under the curve values, ranging from 0.73–0.80 for 3-day, 7-day, and 4-week self-reported pill count; specificity values ranged from 0.63–0.78 and sensitivity values ranged from 0.82–0.94. Using this cut-point, adherence was high (88%). However, plasma varenicline levels were weakly correlated with 3-day and 4-week pill count and total pill count (12 weeks) was not correlated with plasma varenicline levels. Patients with head and neck cancer, gastrointestinal cancer, and more advanced disease showed lower varenicline adherence and lower plasma varenicline.

**Conclusions:**

Using the 4 ng/ml cut-point, this study suggests validity of short-term self-reported varenicline adherence among cancer patients undergoing tobacco dependence treatment in contrast to studies in the general population, which supported 12-week pill count.

## Highlights

• First study of the validity of self-reported varenicline use among cancer patients
• Identified cut-point for varenicline plasma adherence
• Supports the validity of short-term self-reported varenicline adherence
• Cancer-related variables influence varenicline adherence
• Varenicline adherence may be higher among cancer patients

## 1 Introduction

Varenicline is one of the most effective medications for tobacco dependence (Cahill, Stevens, Perera, & Lancaster, 2013) even among smokers with psychiatric (Anthenelli et al., 2016) and medical (Price et al., 2017) comorbidities. However, in general population clinical trials, adherence to varenicline rarely exceeds 60% (e.g., Peng et al., 2017), with little known about adherence rates in populations with comorbidities (Pacek, McClernon, & Bosworth, 2017). Across numerous studies, suboptimal adherence significantly reduces the likelihood of successful quitting (Pacek et al., 2017; Peng et al., 2018). Consequently, there is growing recognition for the need to develop interventions to increase varenicline adherence. To do so, however, requires valid methods for assessing varenicline adherence.

Despite numerous clinical studies of varenicline, the literature has relied upon self-reported pill count data to determine varenicline adherence with only two exceptions (Buchanan et al., 2012; Peng et al., 2017). Unfortunately, self-reported pill count data are susceptible to response bias and misreporting, which can overestimate adherence (Dunbar-Jacob & Rohay, 2016). While the two studies that have used a biological assay to measure varenicline adherence provide important information about the validity of self-reported varenicline adherence, extension of these results to include important clinical populations, like cancer patients, is needed.

Upwards of 50% of cancer patients who were smokers prior to their diagnosis continue to smoke after diagnosis (Land et al., 2016) and the US Surgeon General concluded that continued smoking by cancer patients is causally associated with a worse cancer prognosis (U.S. Department of Health and Human Services, 2014). Further, relative to the general population of smokers, cancer patients using varenicline may face greater challenges with adherence because of additional medications and treatments for their cancer that already challenge compliance (Kavookjian & Wittayanukorn, 2015; Sawesi, Carpenter, & Jones, 2014). Varenicline's primary side effects, such as nausea, may exacerbate side effects that cancer patients experience, which reduce medication compliance (Roeland, Aapro, & Schwartzberg, 2015). Moreover, given the stigma associated with smoking after a cancer diagnosis (Riley, Ulrich, Hamann, & Ostroff, 2017), patients may be more likely to overstate their level of adherence to medication. Alternatively, to the extent that cancer patients are more motivated to quit smoking, they may be more adherent to medication.

This study compared self-reported pill count measures of varenicline adherence to plasma varenicline levels in cancer patients undergoing tobacco cessation treatment. Participant characteristics related to varenicline adherence were also assessed. Through assessing the validity of self-report measures of varenicline adherence in this important clinical population, we might be able to identify patients who need medication adherence support.

## 2 Methods

### 2.1 Participants

Participants were enrolled in a National Cancer Institute-funded randomized clinical trial comparing 12 weeks of varenicline + 12 weeks of placebo to 24 weeks of varenicline (NCT01756885). Only data from the 12-week open-label treatment phase were used for this study. To be eligible for the trial, participants were required to be >age 18 and to have: received a cancer diagnosis or cancer treatment within the past 5 years, reported daily smoking, and reported an interest in quitting smoking. Additional eligibility criteria and exclusion criteria are described elsewhere (Miele et al., 2018; Price et al., 2017). For this study, data from 76 participants who provided blood for varenicline testing were used (only participants from the University of Pennsylvania site were asked to provide samples due to budget constraints). Of the sample characteristics (Table 1), participants who provided a sample had a higher disease stage and carbon monoxide (CO) at study entry (*p*'s < 0.05), vs. participants who did not provide a sample.

**Table 1:** Characteristics of sample and difference between adherent and non-adherent participants based on plasma varenicline cut-point of 4.0 ng/ml.

Characteristic	Adherent (*N* = 67) (>4 ng/ml, *N* = 67)	Non-adherent (*N* = 9) (NN = (≤4 ng/ml, *N* = 9)	Total (*N* = 76)	*p*
Sex				0.384
Female	27 (40.3%)	5 (55.6%)	32 (42.1%)	
Male	40 (59.7%)	4 (44.4%)	44 (57.9%)	
Race				0.275
Caucasian	49 (73.1%)	5 (55.6%)	54 (71.1%)	
People of color	18 (26.9%)	4 (44.4%)	22 (28.9%)	
Ethnicity				0.599
Hispanic/Latino	2 (3.0%)	0 (0.0%)	2 (2.6%)	
Not Hispanic/Latino	65 (97.0%)	9 (100.00%)	74 (97.4%)	
Marital Status				0.985
Married	37 (55.2%)	5 (55.6%)	42 (55.2%)	
Not married	30 (44.8%)	4 (44.4%)	30 (44.8%)	
Education				0.103
Below college graduate	41 (61.2%)	8 (88.9%)	49 (64.5%)	
College graduate or beyond	26 (38.8%)	1 (11.1%)	27 (35.5%)	
Income				0.564
<20,000	9 (13.6%)	2 (22.2%)	11 (14.7%)	
20,000 < 75,000	31 (47.0%)	5 (55.6%)	36 (48.0%)	
>75,000	26 (39.4%)	2 (22.2%)	28 (37.3%)	
Employment				0.66
Employed	32 (47.8%)	5 (55.6%)	37 (48.7%)	
Not employed	35 (52.2%)	4 (44.4%)	39 (51.3%)	
Tumor type				**0.045**
Head and neck	4 (7.3%)	2 (25.0%)	6 (9.5%)	
Lung	8 (14.5%)	0 (0.0%)	8 (12.7%)	
Hematological	7 (12.7%)	0 (0.0%)	7 (11.1%)	
Breast	6 (10.9%)	0 (0.0%)	6 (9.5%)	
Gastrointestinal	2 (3.6%)	2 (25.0%)	4 (6.3%)	
Genitourinary	13 (23.6%)	4 (50.0%)	17 (27.0%)	
Skin	13 (23.6%)	0 (0.0%)	13 (20.6%)	
Kidney, pancreas, and liver	2 (3.6%)	0 (0.0%)	2 (3.2%)	
Cancer stage				**0.005**
Stage 0–2	6 (8.8%)	0 (0%)	6 (7.9%)	
Stage 3–4	10 (14.7%)	6 (75%)	16 (21.1%)	
Remission	18 (26.5%)	1 (12.5%)	19 (25.0%)	
Stage not specified	34 (50.0)	1 (12.5%)	35 (46.1%)	
Age (mean, SD)	59.60 (9.097)	57.44 (6.821)	59.34 (8.848)	0.497
Cigarettes per Day (mean, SD)	15.87 (8.467)	13.00 (5.657)	15.53 (8.208)	0.329
FTCD^a^ (mean, SD)	4.40 (2.236)	3.89 (1.900)	4.34 (2.194)	0.513
CO at Intake (mean, SD)	18.13 (10.868)	20.00 (10.283)	18.36 (10.751)	0.628
Karnofsky Score (mean, SD)	90.31 (11.948)	91.25 (9.910)	90.42 (11.681)	0.832
ECOG Score (mean, SD)	0.38 (0.506)	0.00 (0.000)	0.31 (0.479)	0.221
Age started smoking (mean, SD)	16.55 (6.048)	16.11 (2.619)	16.50 (5.740)	0.830
Years smoked (mean, SD)	41.81 (10.851)	41.22 (6.648)	41.74 (10.409)	0.876

Bold numbers indicate significant differences between adherent and non-adherent participants.

a Fagerstrom test of cigarette dependence.

### 2.2 Procedures

The IRBs at the University of Pennsylvania, Northwestern University, and the University of Toronto (which analyzed the blood samples for varenicline levels) approved this study and informed consent was obtained. Following telephone and in-person screening, eligible participants were randomized to 12 vs. 24-weeks of varenicline. Varenicline was provided as per FDA guidelines and all participants received 5 behavioral smoking cessation counseling sessions. Assessments were conducted in-person at Weeks 0 (initiation of medication), 4, and 12. A blood sample (10 ml) was collected at Week 4 from 76 Penn participants who attended the visit; 38 participants either refused the blood draw or did not complete the session in person. Blood was drawn into a tube containing ethylenediaminetetraacetic acid, immediately iced, centrifuged at 4 °C to separate the plasma, and were analyzed in Dr. Tyndale's laboratory following established methods (Peng et al., 2017).

### 2.3 Measures

#### 2.3.1 Covariates

Demographic, cancer-related (e.g., tumor site/stage), and smoking data were gathered during screening. CO was collected and tobacco dependence was assessed using the Fagerström Test for Cigarette Dependence (FTCD; Heatherton, Kozlowski, Frecker, & Fagerström, 1991).

#### 2.3.2 Pill count measures

Self-reported varenicline adherence was assessed using timeline follow-back (TLFB; Brown, Burgess, Sales, Evans, & Miller, 1998), with participants reporting the number of pills taken each day since the previous visit and returning medication blister packs. Pill count adherence measures were created by dividing the reported number of pills taken by the total number of prescribed pills for each time period (3-day, 7-day, 4-week, 12-week). The 3-day, 7-day, and 4-week pill counts refer to the number of prescribed pills taken during the respective time-frames prior to plasma sample acquisition (Week 4); 12-week pill count is the total number of pills prescribed. Consistent with FDA guidelines for 12 weeks of varenicline treatment, a total of 165 pills were prescribed; for 3-day, 7-day, and 4-week adherence, the prescribed number of pills were 6, 14, and 53 pills, respectively.

#### 2.3.3 Plasma varenicline levels

Plasma samples were collected 4 weeks after initiating treatment (Week 4). Varenicline levels were determined using liquid chromatography-tandem mass spectrometry (Peng et al., 2017). Samples were collected at this time point because it was the first in-person visit when therapeutic levels of varenicline would be reached.

### 2.4 Data analysis

We followed procedures used in Buchanan et al. (2012) to determine a cut-point for plasma varenicline that differentiated adherent vs. non-adherent participants for the four pill count measures using Receiver Operating Characteristic (ROC) curve analyses. While Buchanan et al. (2012) used 2.0 ng/ml as the cut-point (and Peng et al., 2017 used 4.7 ng/ml, adjusted for saliva vs. plasma), we used the same exploratory approach as Buchanan et al. (2012) to determine a cut-point in this sample given use of a clinical population which may differ in important ways from the general population of smokers studied in Buchanan et al. (2012) and Peng et al. (2017). Using ROC analyses, we examined 2.0, 4.0, 6.0, and 8.0 ng/ml as potential cut-points for adherence using plasma varenicline. Using this approach, we determined how cut-points for plasma varenicline differentiate adherence, which is captured by area under the curve (AUC) values. When the AUC value equals 1.0, the cut-point offers perfect differentiation, but AUC values of >0.70 are acceptable; AUC values are evaluated using probability testing and 95% confidence intervals.

Next, with a cut-point determined by AUC values, positive and negative predictive value estimates were calculated (with 95% confidence intervals) to assess the accuracy of pill count data vs. plasma varenicline, and we described the sensitivity and specificity of each self-report measure. We used Pearson correlation to assess the relationship between self-reported pill count measures of varenicline adherence and plasma varenicline. Lastly, we examined differences in demographic, cancer-related, and smoking variables between adherent and non-adherent participants based on varenicline levels utilizing chi-square and ANOVA. We also used multiple regression, with tertiles for plasma varenicline, to examine correlates of varenicline adherence.

## 3 Results

The mean 3-day pill count was 5.6 (SD = 1.33), the mean 7-day pill count was 13.1 (SD = 2.55), the mean 4-week pill count was 50.8 (SD = 6.1), the mean 12-week pill count was 138.42 (SD = 40.3), and the mean plasma varenicline was 7.27 ng/ml (SD = 3.6).

### 3.1 ROC analyses

**Fig. 1:**
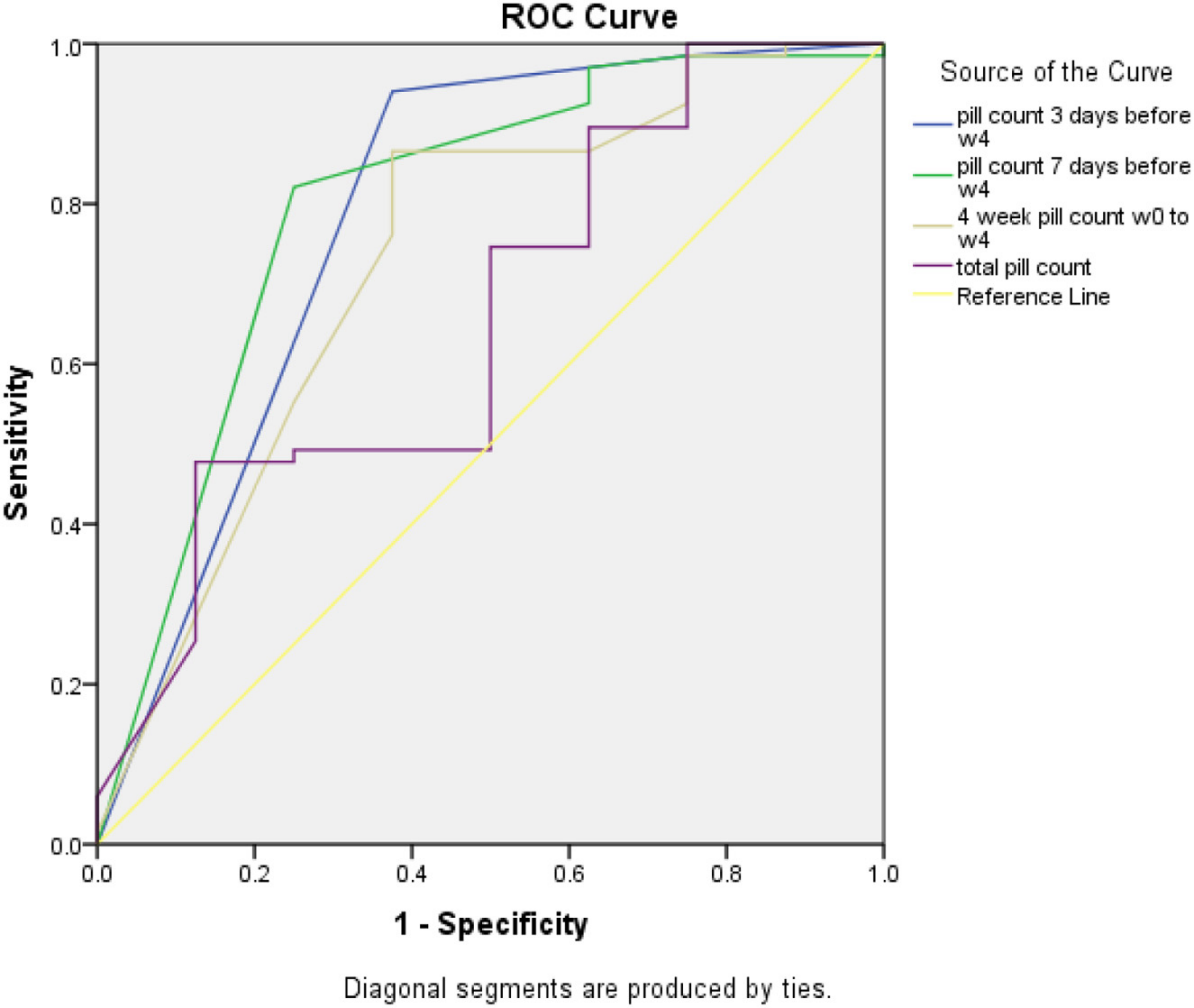
Receiver operating characteristic (ROC) curve of pill counts in discriminating adherence as defined by a biological measure (4.0 ng/ml varenicline cut point).

A plasma varenicline cut-point of 2.0 ng/ml to define adherence yielded AUC values from 0.68 for 3-day pill count (*p* = 0.39) to 0.84 for 7-day pill count (*p* = 0.11), but none of the pill-count AUC values were statistically significant. Using a plasma varenicline cut-point of 6.0 or 8.0 ng/ml yielded AUC values <0.57 for all pill count measures (*p*'s > 0.05). Using a plasma varenicline cut-point of 4.0 ng/ml to define adherence (Fig. 1), 7-day pill count (AUC = 0.79) and 3-day pill count (AUC = 0.76) had AUC values that were statistically significant (*p*'s < 0.03). The 4-week pill count measure had an acceptable AUC value for 4.0 ng/ml (0.71; *p* = 0.07). The optimal cut-point of pill counts for discriminating adherence based on 4.0 ng/ml plasma varenicline levels ranged from 83% to 93%. The probability that pill counts correctly predicted adherence defined by varenicline plasma (i.e., true positives) ranged from 0.95–0.97, and was higher than the probability that pill counts predicted non-adherence (i.e., true negatives), which ranged from 0.19–0.58 (Table 2).

**Table 2:** Measures of the relationship between varenicline adherence self-report and varenicline plasma levels with cut-point of 4.0 ng/ml.

	AUC (95% CI); *p*-value	Cutpoint, %	Sensitivity, % (95% CI)	Specificity, % (95% CI)	PPV % (95% CI)	NPV % (95% CI)
3-Day pill count (0–6)	0.79 (0.58, 1.00); **0.009**	0.83 (5/6)	0.94 (0.85–0.98)	0.63 (0.25–0.92)	0.95 (0.87–0.99)	0.58 (0.24–0.87)
7-Day pill count (0–14)	0.80 (0.61, 0.99); **0.006**	0.93 (13/14)	0.82 (0.71–0.90)	0.78 (0.40–0.97)	0.96 (0.88–1.00)	0.37 (0.17–0.62)
4-week pill count (0–56)	0.73 (0.52, 0.94); **0.04**	0.89 (50/56)	0.87 (0.76–0.94)	0.67 (0.30–0.93)	0.95 (0.86–0.99)	0.40 (0.17–0.68)
Total pill count (0–177)	0.66 (0.45, 0.87); 0.15	0.92 (162/177)	0.48 (0.35–0.60)	0.89 (0.52–1.00)	0.97 (0.84–1.00)	0.19 (0.09–0.34)

Bold numbers indicate significant AUC values.

### 3.2 Correlations between varenicline pill count and varenicline levels

The pill count measures examined (3-day, 7-day, 4-week, and 12-week) were significantly correlated with each other (Spearman's rhos of 0.45–0.83, *p*'s < 0.001), but weakly correlated with plasma varenicline (Spearman's rhos of −0.02-0.24, *p*'s of 0.04–0.90; Table 3).

**Table 3:** Correlations between self-reported varenicline pill count and varenicline plasma levels.

Measures of adherence	Plasma varenicline (ng/ml)	3-Day pill count	7-Day pill count	14-Day pill count	12-Week pill count
Plasma varenicline (ng/ml)	1	–	–	–	–
3-Day Pill Count	0.23 (**0.05**)	1	–	–	–
7-Day Pill Count	0.21 (0.07)	0.83 (<0.001)	1	–	–
4-Week Pill Count	0.24 (**0.04**)	0.58 (<0.001)	0.68 (<0.001)	1	–
Total Pill Count	−0.02 (0.90)	0.45 (<0.001)	0.48 (<0.001)	0.75 (<0.001)	1

Note. Spearman correlation coefficients are displayed. *p*-Values (2-tailed) are indicated in brackets. Higher values reflect greater adherence.

Bold numbers indicate significant correlations between plasma varenicline and self-report measure.

### 3.3 Correlates of varenicline adherence

Higher rates of non-adherence were reported by patients with head and neck or gastrointestinal cancer and by patients with stage 3 or 4 disease (*p*'s < 0.05; Table 1). In a multiple regression model predicting varenicline levels, later disease stage was associated with lower varenicline levels (b = 0.30, *p* = 0.02), when controlling for tumor type and sex.

## 4 Discussion

Developing and implementing effective tobacco dependence treatments for cancer patients requires valid assessments of medication adherence to target patients who experience difficulty with adherence with supplemental interventions. This is the first study to explore the validity of self-report measures of varenicline adherence among cancer patients.

Using a cut-point for plasma varenicline of 4.0 ng/ml, 3-day, 7-day, and 4-week pill count – measures more proximal to the plasma collection – had the strongest association with plasma varenicline. The AUC values for each measure were significant and were >0.73, and the sensitivity values were >0.82 and the specificity values were >0.67. As with a varenicline adherence study with African American smokers who did not have cancer (Buchanan et al., 2012), these results support use of these proximal self-report measures of varenicline adherence. However, this is in contrast to our past study (Peng et al., 2017), which found that 12-week pill was the preferable measure of varenicline adherence. Differences in the samples (a cancer diagnosis) and in the biosample collected to assess varenicline levels (plasma vs. saliva) could account for this difference. Further, while the relationship between 3-day and 4-week pill count and varenicline levels was significant in this study, the strength of the relationship was modest and 7-day and 12-week pill count data were not significantly associated with varenicline levels. Peng et al. (2017) and Buchanan et al. (2012) both reported correlations with varenicline levels and 3-day pill count but, Peng et al. (2017) found a significant association between 12-week pill count and varenicline levels. Again, difference across the sample may help explain variability in these relationships across studies.

Lastly, we did not find demographic or smoking-related characteristics to be associated with varenicline adherence. While relatively few studies have examined correlates of varenicline adherence, previous studies have reported that lower adherence is associated with female gender, younger age, less education, non-white race, and higher tobacco dependence (Pacek et al., 2017). In the present study, we found that patients with head and neck cancer or gastrointestinal cancer were more likely to be non-adherent and to have lower varenicline levels. Consistent with a previous study (Schnoll et al., 2004), head and neck cancer patients may manifest additional barriers to successful smoking cessation, including lower varenicline adherence, given perceptions of a more favorable prognosis. Likewise, gastrointestinal cancer patients may experience greater challenges with adherence given perceptions that their cancer is less associated with tobacco use. Further, the results also indicate that patients with more advanced stage disease were more likely to be non-adherent. We have previously found that advanced cancer stage is associated with lower confidence in quitting smoking (Martinez et al., 2009), which may manifest itself in lower varenicline adherence. As such, from a clinical perspective, these sub-groups of patients may require additional support to ensure that they achieve adequate adherence and benefit from the use of varenicline.

These results should be considered in the context of study limitations, which include a small sample size, a single assessment of plasma varenicline, and limited generalizability from using a sample selected based on inclusion and exclusion criteria. Also, since this study used a cut-point for plasma varenicline adherence that was different from previous studies (given differences in sample characteristics), these findings should be considered exploratory and in need of replication. Indeed, the rate of varenicline adherence found in the present sample using plasma (~88%) is considerably higher than in studies with the general population (Buchanan et al., 2012; Peng et al., 2017), even when considering the cut-point used in those trials; differences between a population with a serious tobacco-related medical comorbidity and the general population may influence varenicline adherence and the relationship between self-reported and biological measures of varenicline use (which was modest but higher in this population). Nevertheless, this is only the third study of the relationship between self-reported and a biological assay of varenicline use and the first study to do so among cancer patients who may have unique patterns of adherence. Overall, the findings suggest acceptable validity using short-term self-report measures of varenicline adherence among cancer patients, which can be important for future studies and interventions with this population.
